# Neurocognitive development in children and their online and offline self-appraisals

**DOI:** 10.1192/j.eurpsy.2021.583

**Published:** 2021-08-13

**Authors:** G. Soldatova, E. Rasskazova, V. Sadovnichaja

**Affiliations:** 1 Faculty Of Psychology, Lomonosov Moscow State University, Moscow, Russian Federation; 2 Clinical Psychology, Moscow State University, Moscow, Russian Federation

**Keywords:** Internet, adolescents, neurocognitive development

## Abstract

**Introduction:**

Internet is an important sphere of activity in children 7-11 years old (Finkelhor et al., 2014; Li et al., 2015; Nasi, Koivusilta, 2013) creating a sphere of possible mental health risks (Livingstone et al., 2011). Neurocognitive deficiency could increase these risks online due to control and change replies and activities online.

**Objectives:**

The aim was to study relationship between neurocognitive functioning in children 7-11 years old and their self-appraisals online and offline.

**Methods:**

50 children 7-11 years old (primary school in Russia, 25 males and 25 females) participated in neuropsychological observation (Akhutina, 2016) and filled Dembo-Rubinstein scales of self-appraisals both for Internet and offline (used descriptors: healthy, happy, clever, kind, confident, independent, Cronbach’s alpha=.63-.65).

**Results:**

Difficulties in the functions of programming and control, serial organization, auditory-speech processing are related to better self-appraisals online (r=.30-.35, p<.01) but not offline. Difficulties in functions of the right hemisphere are more strongly related to online self-appraisals (r=.51) than to offline self-appraisals (r=.31). Adjusting for offline self-appraisals, serial organization, auditory-speech processing and functions of the right hemisphere predict difference in offline and online self-appraisals (ΔR²=6.6-13.0%, p<.05).

**Conclusions:**

Neurocognitive deficiency in children 7-11 years old could lead to unrealistic appraisals of themselves online but not offline increasing probability of problem behavior in the Internet. Study is supported by the Russian Foundation for Basic Research, project 19-29-14181mk.

**Conflict of interest:**

Study was supported by the Russian Foundation for Basic Research, project 19-29-14181mk. There are no other significant relationships.

